# *Runx1* is sufficient but not required for cardiomyocyte cell-cycle activation

**DOI:** 10.1152/ajpheart.00782.2023

**Published:** 2024-06-07

**Authors:** Kaelin A. Akins, Michael A. Flinn, Samantha K. Swift, Smrithi V. Chanjeevaram, Alexandra L. Purdy, Tyler Buddell, Mary E. Kolell, Kaitlyn G. Andresen, Samantha Paddock, Sydney L. Buday, Matthew B. Veldman, Caitlin C. O’Meara, Michaela Patterson

**Affiliations:** ^1^Department of Cell Biology, Neurobiology, and Anatomy, Medical College of Wisconsin, Milwaukee, Wisconsin, United States; ^2^Cardiovascular Center, Medical College of Wisconsin, Milwaukee, Wisconsin, United States; ^3^Department of Physiology, Medical College of Wisconsin, Milwaukee, Wisconsin, United States

**Keywords:** cardiomyocyte, endomitosis, ploidy, proliferation, Runx1

## Abstract

Factors responsible for cardiomyocyte proliferation could serve as potential therapeutics to stimulate endogenous myocardial regeneration following insult, such as ischemic injury. A previously published forward genetics approach on cardiomyocyte cell cycle and ploidy led us to the transcription factor, *Runx1*. Here, we examine the effect of *Runx1* on cardiomyocyte cell cycle during postnatal development and cardiac regeneration using cardiomyocyte-specific gain- and loss-of-function mouse models. RUNX1 is expressed in cardiomyocytes during early postnatal life, decreases to negligible levels by 3 wk of age, and increases upon myocardial injury, all consistent with observed rates of cardiomyocyte cell-cycle activity. Loss of *Runx1* transiently stymied cardiomyocyte cell-cycle activity during normal postnatal development, a result that corrected itself and did not extend to the context of neonatal heart regeneration. On the other hand, cardiomyocyte-specific *Runx1* overexpression resulted in an expansion of diploid cardiomyocytes in uninjured hearts and expansion of 4 N cardiomyocytes in the context of neonatal cardiac injury, suggesting *Runx1* overexpression is sufficient to induce cardiomyocyte cell-cycle responses. Persistent overexpression of *Runx1* for >1 mo continued to promote cardiomyocyte cell-cycle activity resulting in substantial hyperpolyploidization (≥8 N DNA content). This persistent cell-cycle activation was accompanied by ventricular dilation and adverse remodeling, raising the concern that continued cardiomyocyte cell cycling can have detrimental effects.

**NEW & NOTEWORTHY** *Runx1* is sufficient but not required for cardiomyocyte cell cycle.

Listen to this article’s corresponding podcast at https://ajpheart.podbean.com/e/runx1-drives-cardiomyocyte-cell-cycle-activation/.

## INTRODUCTION

*Runx1* is a transcription factor with well-characterized roles in hematopoiesis (1, 2). The cardiac regeneration field has identified it as a possible marker of cardiomyocyte (CM) “dedifferentiation” (3, 4), a cellular process whereby fetal genes are reactivated and sarcomeres are disassembled to support cardiomyocyte proliferation (5). However, little is known about the functional role of *Runx1* in cardiomyocyte cell cycle during development or regeneration. Our laboratory recently identified *Runx1* from a previously published forward genetic screen that identified genes associated with cardiomyocyte cell cycle and ploidy (6, 7). Swift et al (6) determined that cardiomyocyte-specific overexpression of *Runx1* was sufficient to drive cardiomyocyte cell-cycle entry and proliferation, both in the context of postnatal development and adult ischemic injury. However, the requirement for endogenous *Runx1* in cardiomyocyte proliferation was not tested.

The first week of neonatal life in the mouse presents a unique opportunity for examining cardiomyocyte proliferation. *Postnatal* (P) *day 1* mice are competent to regenerate their hearts following myocardial injury, whereas this regenerative potential is lost by P7 (8). Here, we use a cardiomyocyte-specific *Runx1* loss-of-function mouse model (1) in a regeneration-competent setting to examine the requirement for *Runx1* in neonatal cardiomyocyte proliferation. *Runx1* overexpression from birth is sufficient to drive cardiomyocyte proliferation and expand the number of diploid cardiomyocytes (6), a population believed to have retained proliferative competence (7). Thus, we further test if *Runx1* overexpression can extend the neonatal regenerative window. Our present studies demonstrate that endogenous *Runx1* expression in cardiomyocytes is associated with cell-cycle reentry and while loss of *Runx1* can modestly suppress cardiomyocyte cell cycle, the effects are transient. Furthermore, there is no evidence that loss of *Runx1* impacts cardiomyocyte proliferation in the context of neonatal cardiac injury. Overexpression of *Runx1* can drive cardiomyocyte cell-cycle reentry; however, continued expression for 1 mo results in dramatic increases in cardiomyocyte polyploidy and impaired ventricular function independent of injury status.

## METHODS

### Mice

Animals were housed in the Medical College of Wisconsin’s Biomedical Research Center Animal Facility. All procedures were performed under the approval and supervision of the local Institutional Animal Care and Use Committee. *Runx1* conditional knockout mouse (1) and *Runx1* conditional overexpression mouse (2) were kind gifts from the laboratory of Dr. Nancy Speck. C57Bl/6J (JAX Stock No. 000664) and Myh6-Cre driver mice (JAX Stock Nos. 011038 and 005657) were purchased from Jackson Laboratories. All mice have been backcrossed and maintained on a C57Bl/6J background and both sexes were used in all experiments. Sex for overexpression studies were compared indicated by blue (male) and red (female) data points, and no significant differences or trends distinguished the two sexes, thus sexes were then pooled for all experiments. Successful excision of the cKO allele was confirmed by a three-primer PCR as described by Growney et al. (1).

### Neonatal Mouse Surgeries

P1 ventricular resection was performed as described by Paddock et al. (9) and Mahmoud et al. (10). Briefly, P1 neonates were anesthetized on ice for ∼2 min. An incision was created between the third and fourth intercostal ribs and pressure was applied to pop the heart out. The surgeon removed ∼10–15% of the ventricular apex using microspring scissors. Hearts were returned to the thoracic cavity, muscle layer was closed with 6-0 prolene suture, and skin was sealed with Vet Bond. P6 myocardial infarctions were performed as described by Paddock et al. (9) and Mahmoud et al. (10). Briefly, P6 neonates were anesthetized on ice for ∼2–3 min and heart was popped out of the thoracic cavity as described earlier. A 7-0 prolene suture was passed under the left anterior descending artery and a double knot was tied and heart was returned to thoracic cavity. Uninjured, sham animals did not receive any surgical procedures.

### Echos

Vevo 3100 Series, X550 probe was used to capture cardiac echoes from neonatal mice at 21 days post-resection (DPR; Runx1 KO neonates) and 28 days postinfarction (DPI) or P35 sham (Runx1 OE neonates) under light anesthesia (∼2% isoflurane). Videos were taken in long-axis (LAX) B-mode to measure ventricular luminal area and ejection fraction. M-mode measurements were used to capture posterior wall thickness.

### EdU Injections

5-Ethynyl-2′-deoxyuridine (EdU, Thermo Fisher, E10187) was dissolved in DMSO at 100 mg/mL to create a stock solution. The stock solution was aliquoted and stored at −20°C. Immediately before injection, the stock solution was thawed and further diluted to 1 mg/mL with sterile PBS. Neonatal mice were injected with 10 mg/kg EdU by subcutaneous injection at the times indicated in the respective figures.

### Tamoxifen Administration

Tamoxifen (10 mg; Sigma, T5648) was dissolved in 100 µL of prewarmed 100% ethanol and then diluted in 1.9 mL of prewarmed sunflower oil to a final working concentration of 5 mg/mL. Neonates were injected subcutaneously at P0 and P1 with 50 mg/kg of the tamoxifen solution (∼10 µL into a 1-g pup).

### Fixation and Histology

Hearts were dissected from animals and hung on a Langendorff apparatus. Hearts were first perfused with calcium-free Tyrode’s to clear the heart of blood, then 5 mL of 4% paraformaldehyde (PFA). Perfused hearts were further fixed overnight in 4% PFA at 4°C. Following fixation, hearts were processed by the local histology core in progressive ethanol and xylene steps before being embedded in paraffin molds. Resected hearts were oriented for frontal four-chamber view while myocardial infarction (MI) hearts were oriented for coronal two-chamber view. Sections (4 µM) were collected every 200 µM creating a series across the whole heart.

### Trichrome

Sectioned tissue was warmed in an oven at 60°C for 30 min before undergoing deparaffinization in xylene (5 min) and tissue rehydration with decreasing ethanol (EtOH) concentrations (100% EtOH for 5 min, 95% EtOH for 5 min, 70% EtOH for 5 min, and diH_2_O for 3 times for 1 min each). Slides were then placed in Bouin’s fixative (Leica, 38016SS1A) for 60 min at 60°C and washed in 20°C tap water for 30–45 min. Slides were then stained with hematoxylin solution (Sigma, H3136 and F1513) for 30 min and Gomori’s Trichrome (Leica, 38016SS2E) for 30 min according to manufacturer’s protocol. Slides were rinsed in 0.05% acetic acid four times for 1 min each and washed in diH_2_O. Slides were dried overnight and coverslipped with Permount (Fisher, SP15).

### Immunofluorescence

Sections were deparaffinized and rehydrated as described above in Trichrome staining methodology. Antigen retrieval was performed using freshly made pH 6.0 sodium citrate buffer, consisting of 1.98-g sodium citrate dihydrate (Sigma, W302600), 1-mL Triton-X-100 (Sigma, T9284), and 0.5-mL Tween-20 (Fisher, BP337) dissolved in 1 L of ddH_2_O, warmed to 95–100°C in a vegetable steamer for 35 min. Slides were allowed to cool in the same citrate buffer for 20 min before washing in PBS. Slides were blocked for 1 h at room temperature (RT) in 5% normal donkey serum (Jackson Immuno Research, 017-000-121) and 5% bovine serum albumin (Sigma, A9647) dissolved in PBS. Primary antibodies were diluted to working concentrations in blocking solution (see Table 1 for complete list of antibodies and concentrations). Slides were incubated with primary solution overnight at 4°C, washed in PBS for 10 min, and incubated with secondary antibodies diluted 1:500 in PBS for 1 h at RT. Secondary antibodies included Alexa fluor donkey-anti-goat 555 (Thermo, A-32816), donkey-anti-rabbit 488 (Thermo, A-21206), donkey-anti-mouse 647 (Thermo, A-31571), and donkey-anti-rat 488 (Thermo, A-150153). EdU click-it reaction Alexa fluor 488 dye (Thermo, C10337) was performed following manufacturer’s protocol. Slides were washed in PBS for 5 min, treated with 0.03% Sudan Black B (Sigma, 199664,) dissolved in 70% EtOH for 20 min, and washed in PBS for 5 min. Slides were stained for 4′,6-diamidino-2-phenylindole (DAPI; 1:1,000 in PBS) for 5 min and washed one final time with PBS for 5 min. Slides were coverslipped with ProLong Gold (Thermo, 36930).

**Table 1.** List of antibodies

Antigen	Working Dilution	Vendor, Cat. No.
Goat anti-Nkx2.5	1:250	Abcam, ab106923
Rabbit anti-Runx	1:200	Abcam, ab92336
Mouse anti-cardiac troponin T	1:250	Abcam, ab8295
Rat anti-Ki67	1:250	Thermo, 14-5698-82
Mouse anti-proliferating cell nuclear antigen	1:1,000	Sigma, P8825
Rabbit anti-PH3	1:250	Millipore, 06-570
Rabbit anti-myosin light chain 2 (Myl2)	1:250	Abcam, ab79935
Mouse anti-α-actinin	1:250	Sigma, A7811-.2ML

### Microscopy and Image Analysis

**Figure 1.**
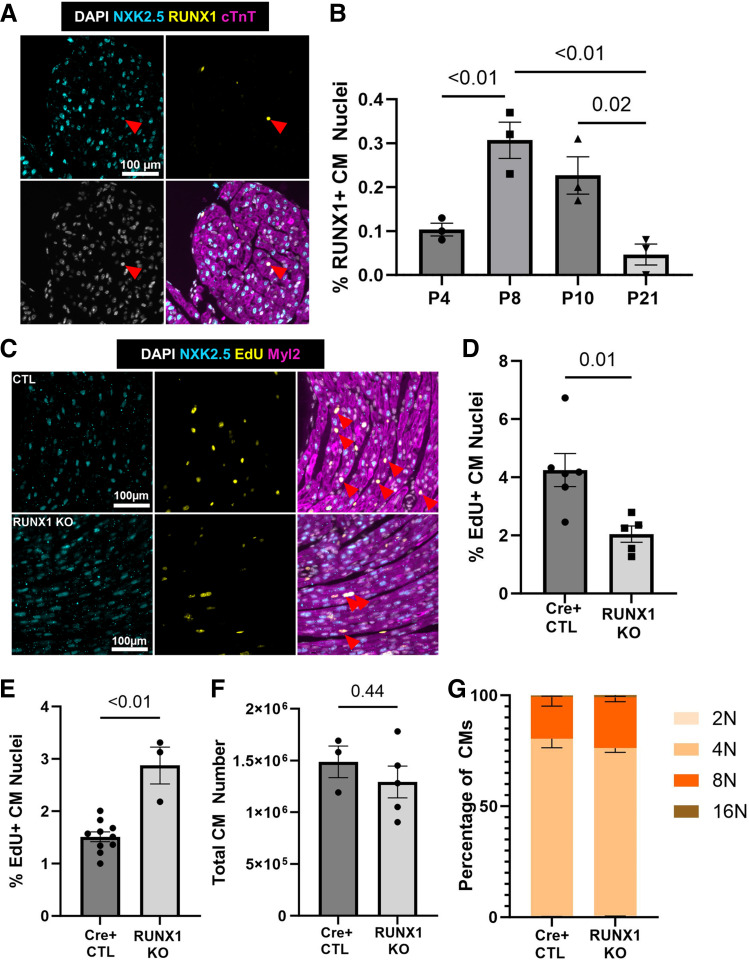
Loss of Runx1 transiently represses cardiomyocyte cell cycle during neonatal development. *A*: representative ×20 image of an immunofluorescence stain for NKX2.5 (cyan), RUNX1 (yellow), cardiac troponin T (cTnt, magenta), and 4′,6-diamidino-2-phenylindole (DAPI; grayscale). Scale bar = 100 µm. *B*: quantification of temporal expression of RUNX1-positive cardiomyocyte (CM) nuclei in C57Bl/6J hearts, P4 (*n* = 3 hearts), P8 (*n* = 3 hearts), P10 (*n* = 3 hearts), and P21 (*n* = 3 hearts). Indicated *P* values were calculated by one-way ANOVA followed by Tukey honest significant difference (HSD) post hoc analysis. Data are means ± SE. *C*: representative ×20 image of an immunofluorescence stain for NKX2.5 (cyan), 5-ethynyl-2′-deoxyuridine (EdU; yellow), myosin light chain 2 (Myl2; magenta), and DAPI (grayscale). Scale bar = 100 µm. *D*: quantification of EdU-positive cardiomyocytes labeled at P7 in RUNX1 knockout (KO) neonates (*n* = 5 hearts) compared with Cre-positive controls (*n* = 6 hearts). *P* value calculated by two-tailed Student’s *t* test. Data are means ± SE. *E*: quantification of EdU-positive cardiomyocytes labeled at P10 in RUNX1 KO neonates (*n* = 3 hearts) compared with Cre-positive controls (*n* = 10 hearts). *P* value calculated by two-tailed Student’s *t* test. Data are means ± SE. *F*: quantification of whole cell cardiomyocyte number at P21 in RUNX1 KO neonates (*n* = 5 hearts) compared with Cre-positive controls (*n* = 3 hearts). *P* value calculated by two-tailed Student’s *t* test. Data are means ± SE. *G*: quantification of 2 N (1 × 2 N), 4 N (sum of 1 × 4 N and 2 × 2 N populations), 8 N [sum of 1 × 8 N, 2 × 4 N, tri (1 × 4 N + 2 × 2 N) and 4 × 2 N populations], and 16 N [sum of 1 × 16 N, 2 × 8 N, tri (1 × 8 N + 2 × 4 N) and 4 × 4 N populations] ploidy classes represented as a percentage of total cardiomyocytes at P21 in RUNX1 KO neonates (*n* = 5 hearts) compared with Cre-positive controls (*n* = 3 hearts). *P* values were calculated by one-way multivariate ANOVA followed by Wilks’ Lambda post hoc test. Reported *P* values are the Wilks’ Lambda post hoc analysis assessing genotype contributions. Data are means ± SE.

**Figure 2.**
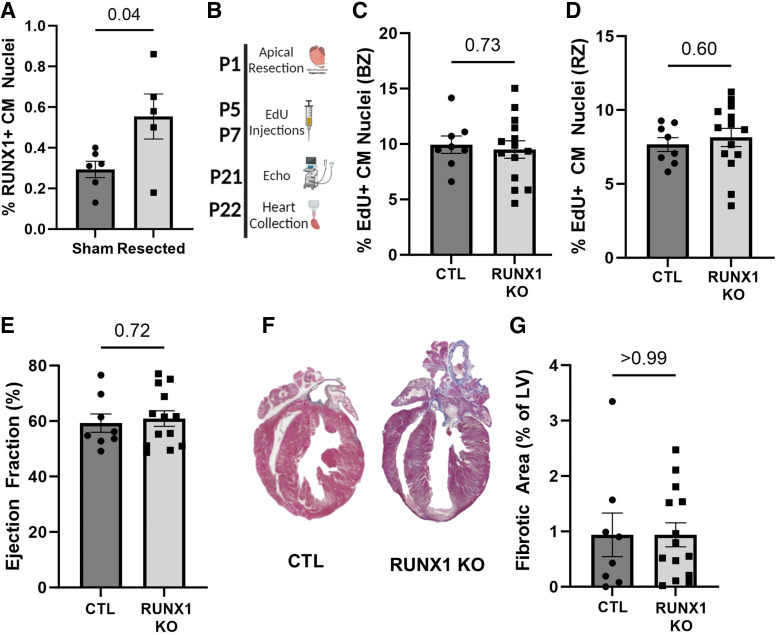
RUNX1 is not required for neonatal cardiac regeneration. *A*: quantification of RUNX1-positive cardiomyocytes at 7 days post-resection (DPR) in C57 sham (*n* = 6) and resected (*n* = 5) neonates. *P* values calculated by two-tailed Student’s *t* test. Data are means ± SE. *B*: schematic of the experimental design for the RUNX1 knockout (KO) neonatal cardiac injury study. *n* = 8 controls (Ctl = *Myh6-Cre*-negative; *Runx1^f/f^*) and *n* = 14 Runx1 KO (*Myh6-Cre*-positive; *Runx1^f/f^*). A mix of both sexes was used. *C* and *D*: quantification of 5-ethynyl-2′-deoxyuridine (EdU)-positive cardiomyocytes labeled at 5 and 7 DPR in the border zone (BZ) and remote zone (RZ), respectively. EdU^+^NKX2.5^+^ cardiomyocyte (CM) nuclei represented as a percentage of total NKX2.5^+^ nuclei. *P* value calculated by two-tailed Student’s *t* test. *E*: quantification of the ejection fraction at 21 days postinjury (DPI) in RUNX1 KO neonates compared with Ctl. *P* value calculated by two-tailed Student’s *t* test. *F*: representative images of Gomori’s Trichrome stain. *G*: quantification of the fibrosis at 21 DPR in RUNX1 KO compared with Ctl neonates. *P* value calculated by two-tailed Student’s *t* test. Data are means ± SE. Some images were created with a licensed version of BioRender.com.

**Figure 3.**
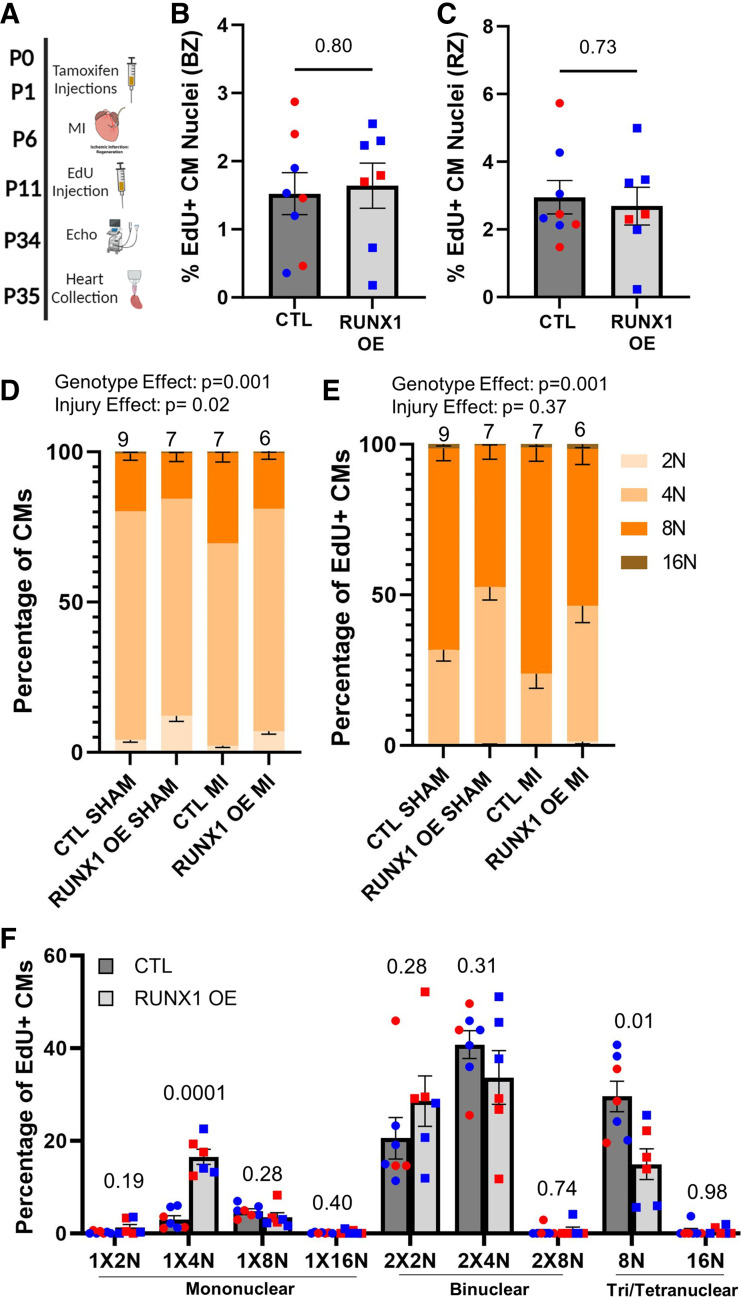
RUNX1 overexpression influences cardiomyocyte (CM) ploidy independent of cardiac injury. *A*: schematic of the experimental design for. *Postnatal day 6* (P6) myocardial infarction (MI) surgeries in RUNX1 overexpression (RUNX1 OE, *n* = 7) neonates compared with Myh6-CreER-positive control (Ctl, *n* = 8). A mix of both sexes was used for this study as indicated by blue (males) and red (females) data points. *B* and *C*: quantification of the 5-ethynyl-2′-deoxyuridine (EdU)-positive cardiomyocyte nuclei labeled at 5 days postinjury (DPI) in the border and remote zones. EdU^+^NKX2.5^+^ CM nuclei are represented as a percentage of total NKX2.5^+^ nuclei. *P* value calculated by two-tailed Student’s *t* test. *D*: quantification of 2 N (1 × 2 N), 4 N (sum of 1 × 4 N and 2 × 2 N populations), 8 N (sum of 1 × 8 N, 2 × 4 N, tri [1 × 4 N + 2 × 2 N] and 4 × 2 N populations), and 16 N (sum of 1 × 16 N, 2 × 8 N, tri [1 × 8 N + 2 × 4 N] and 4 × 4 N populations) ploidy classes represented as a percentage of total cardiomyocytes. Ctl, *Myh6-CreER*-positive animals. RUNX1 OE = *Myh6-CreER*-positive; *Rosa-Runx1^fl/wt^. n* for each group can be found on the graph. Statistical analysis performed by two-way multivariate ANOVA. Reported *P* values are the Wilks’ Lambda post hoc analysis assessing genotype and injury contributions. Further post hoc pairwise comparisons can be found in Supplemental Table S1. *E*: quantification of summed ploidy classes as in *D* in EdU-positive cardiomyocytes labeled at 5 days postinjury (DPI) at sham and 7 DPI in RUNX1 OE animals compared with Ctls. Statistical analysis was performed by two-way multivariate ANOVA. Reported *P* values are the Wilks’ Lambda post hoc analysis assessing genotype and injury contributions. Further post hoc pairwise comparisons can be found in Supplemental Table S2. *F*: quantification of EdU-positive cardiomyocytes labeled at 5 DPI divided into their respective ploidy classes in RUNX1 OE (*n* = 6) vs. Ctl (*n* = 7) littermates at 7 DPI neonates. *P* value for individual ploidy classes calculated by two-tailed Student’s *t* test, following confirmation of statistical significance by two-way multivariate ANOVA in *E*. Data are means ± SE. Some images were created with a licensed version of BioRender.com.

**Figure 4.**
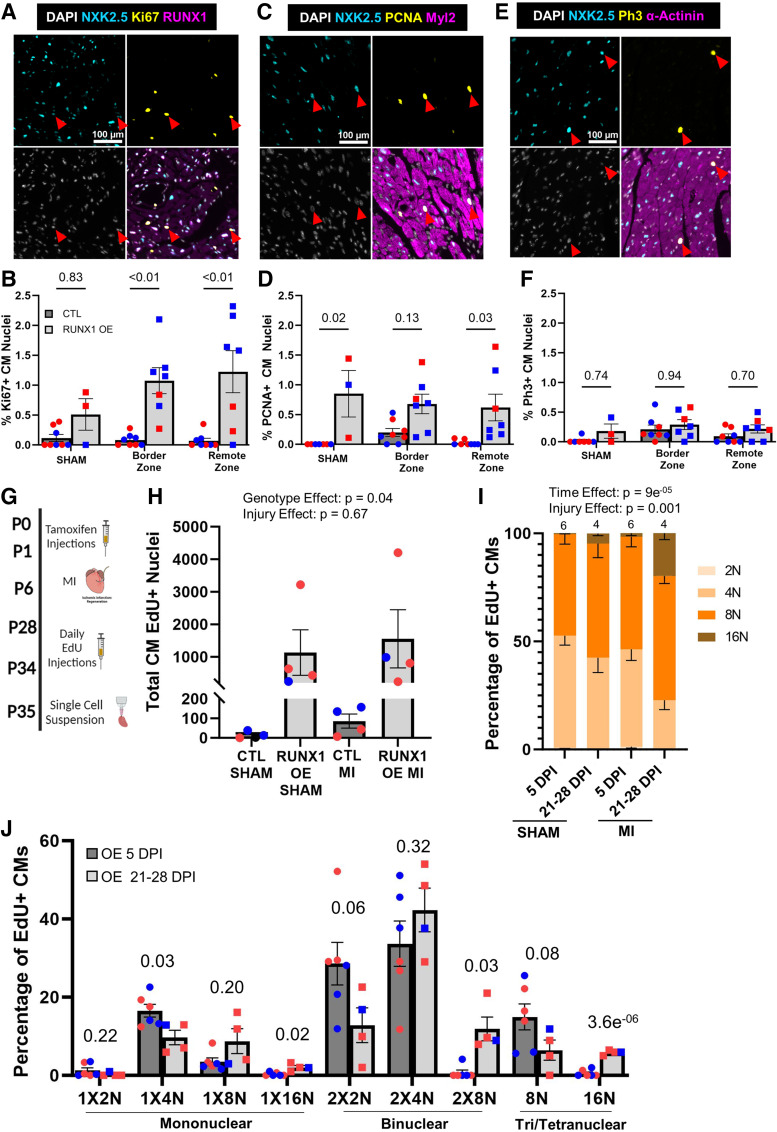
RUNX1 OE increases cardiomyocyte (CM) cell-cycle activity resulting in hyperpolyploidization *A*: representative ×20 image of an immunofluorescence stain for NKX2.5 (cyan), Ki67 (yellow), Runx1 (magenta), and 4′,6-diamidino-2-phenylindole (DAPI; grayscale). Scale bar = 100 µm. *B*: quantification of the Ki67-positive cardiomyocyte nuclei in sham-operated animals [*n* = 6 control (Ctl), 3 RUNX1 OE], and border and remote zones of P6 myocardial infarction (MI)-operated animals assessed 28 days postinjury (DPI; *n* = 8 Ctl, 7 RUNX1 OE). Ki67^+^NKX2.5^+^ CM nuclei are represented as a percentage of total NKX2.5^+^ nuclei. *P* value calculated by two-way ANOVA followed by a Tukey honest significant difference (HSD) post hoc analysis. *C*: representative ×20 image of an immunofluorescence stain for NKX2.5 (cyan), proliferating cell nuclear antigen (PCNA; yellow), Myl2 (magenta), and DAPI (grayscale). Scale bar = 100 µm. *D*: quantification of the PCNA-positive cardiomyocyte nuclei in sham-operated animals (*n* = 6 Ctl, RUNX1 OE), and border and remote zones of *postnatal day 6* (P6) MI-operated animals assessed 28 DPI (*n* = 8 Ctl, 7 RUNX1 OE). PCNA^+^NKX2.5^+^ CM nuclei are represented as a percentage of total NKX2.5^+^ nuclei. *P* value is calculated by two-way ANOVA followed by a Tukey HSD post hoc analysis. *E*: representative ×20 image of an immunofluorescence stain for NKX2.5 (cyan), Ph3 (yellow), α-actinin (magenta), and DAPI (grayscale). Scale bar = 100 µm. *F*: quantification of the Ph3-positive cardiomyocyte nuclei in sham-operated animals (*n* = 6 Ctl, 3 Runx1 OE), and border and remote zones of P6 MI-operated animals assessed 28 DPI (*n* = 8 Ctl, 7 RUNX1 OE). Ph3^+^NKX2.5^+^ CM nuclei are represented as a percentage of total NKX2.5^+^ nuclei. *P* value was calculated by two-way ANOVA followed by a Tukey HSD post hoc analysis. *G*: schematic of the experimental design to assess late-stage cardiomyocyte cell-cycle activity postinjury by single-cell suspension. Tamoxifen delivered as two injections at birth (P0 and P1), MI, or sham operations at P6, 5-ethynyl-2′-deoxyuridine (EdU) injections once daily from 21–28 DPI hearts collected by Langendorff at 29 DPI. *H*: quantification of EdU-positive cardiomyocyte nuclei labeled from 21–28 DPI within single-cell suspensions (*n* = 4 Cre-positive Ctl sham, 4 RUNX1 OE sham, 4 Cre-positive Ctl MI, and 4 RUNX1 OE MI). *P* value was assessed by two-way ANOVA, Wilks’ Lambda values for genotype vs. injury effects are reported on graph. *I*: quantification of summed ploidy classes in EdU-positive cardiomyocytes labeled from 21–28 DPI in RUNX1 OE animals, sham vs. MI, 7 DPI vs. 28 DPI (*n* = 6, 4, 6, 4, as described on graph). *P* value was assessed by two-way multivariate ANOVA. Reported *P* values are the Wilks’ Lambda post hoc analysis assessing genotype and injury contributions. Further pairwise comparisons can be found in Supplemental Table S3. *J*: quantification of EdU-positive cardiomyocytes labeled at 5 DPI and 21–28 DPI divided into their respective ploidy classes in RUNX1 OE 5 DPI (*n* = 6) vs. RUNX1 OE 21–28 DPI (*n* = 4) littermates at 7 DPI neonates. *P* value was calculated by two-tailed Student’s *t* test, following confirmation of statistical significance by two-way multivariate ANOVA (in *I*). Data are means ± SE. Some images were created with a licensed version of BioRender.com.

Images (×20) were captured with a PCO Panda Camera on a Nikon Eclipse 80i fluorescence microscope using NIS Elements software. “Border zone” images were taken in regions that included scar, whereas “remote zone” included areas above the scar or at least 500 µM away from the scar. Equivalent heights of the left ventricle were captured in sham-operated controls. Quantifications were performed by a blinded observer by first identifying all CM nuclei using Nkx2.5 overlap with DAPI and then later determining if the confirmed CM nucleus was also positive for the antigen of interest. Total cardiomyocyte nuclei quantified were experiment dependent. For Fig. 1, *A* and *B*, ∼1,200 cardiomyocytes were quantified per section at P4, ∼700 cardiomyocytes at P8, ∼900 cardiomyocytes at P10, and ∼400 cardiomyocytes at P21 (quantified 3–5 sections). In Fig. 1, *C*–*E*, ∼500 cardiomyocytes were quantified per section for P7 and P10 EdU experiments (quantified 4 sections). In Fig. 2*A*, ∼700 cardiomyocytes were quantified per section for seven DPR experiments (quantified 4 sections). In Fig. 2, *B* and *C*, ∼250–400 cardiomyocytes were quantified per section for border and remote zone for RUNX1 KO EdU experiment (quantified 3–4 sections). In Fig. 3, *B* and *C*, ∼250 cardiomyocytes were quantified per section for border and remote zones for RUNX1 OE EdU experiment (quantified 4 sections). In Fig. 4, *A*–*F*, ∼250 cardiomyocytes were quantified per section for 28 DPI RUNX1 OE cell-cycle marker experiments (quantified 4 sections).

### Single-Cell Suspensions Langendorff, Stain, and Ploidy Analysis

Langendorff single-cell suspensions, stains, and ploidy analysis were performed as described (6). For general ploidy experiments, at least 350 cardiomyocytes were analyzed. For EdU ploidy experiments, at least 80 EdU-positive cardiomyocytes were analyzed.

### RNA Isolation

Runx1 OE neonates along with Myh6-CreER-positive controls were injected with tamoxifen subcutaneously at P0 and P1. Hearts were excised at P10, atria and outflow track removed, and RNA was extracted using TRIzol reagent per manufacturer’s protocol.

### RNA Sequencing and Analysis

RNA samples were sent to BGI Genomics, where they were assessed for RNA quality by bioanalyzer and Oligo dT libraries were prepared. Libraries were sequenced on a DNBSEQ-G400 platform at 150 bp paired-end reads with a total read depth of >20 million reads per sample. Reads were mapped to the mm39 mouse genome using STAR 2.7.8a aligner, quantified to Ensembl transcripts release 110 with Partek E/M, and normalized using the median ratio method. Differential expression analysis was performed by DEseq2. Significantly differentially expressed genes (q value < 0.05, Supplemental Table S4; all Supplemental materials are available at https://doi.org/10.6084/m9.figshare.25769037.v1) were uploaded into Ingenuity Pathway Analysis (IPA), and core analysis was run to identify activated or inhibited pathways and upstream regulators.

### Statistics

All analyses comparing two experimental conditions were assessed for statistical significance by a two-tailed Student’s *t* test using GraphPad Prism. One-way ANOVA (GraphPad Prism) was used for assessing the frequency of Runx1-positive cardiomyocytes across multiple postnatal time points, followed by a Tukey honest significant difference (HSD) post hoc analysis (Fig. 1*B*). In experiments where two independent factors were being compared across multiple experimental groups, SPSS or GraphPad Prism software was used to perform a two-way ANOVA followed by a Tukey HSD post hoc analysis for pairwise comparisons. Finally, SPSS was used to perform a two-way multivariate ANOVA to compare ploidy data where multiple interdependent factors are being assessed as in Figs. 3, *D* and *E*, and 4*I*.

## RESULTS

### Loss of Runx1 Transiently Represses Cardiomyocyte Cell Cycle During Neonatal Development

To first examine the temporal expression of RUNX1 in cardiomyocytes, we immunostained P4, P8, P10, and P21 C57BL/6J hearts and quantified the frequency of RUNX1-positive cardiomyocyte nuclei, as identified by NKX2.5 positivity (Fig. 1*A*). The frequency of RUNX1-positive cardiomyocytes is rare (<0.5% of cardiomyocytes) at all time points; however, they are most prevalent in the second postnatal week when some cardiomyocytes undergo a second round of endomitosis to become 8 N (6). RUNX1 expression then declines to near negligible levels by P21 when most cardiomyocyte cell cycling is complete (Fig. 1*B*). Our previous work described that >80% of RUNX1-positive cardiomyocytes were also Ki67 positive, suggesting a role for *Runx1* in cardiomyocyte cell cycle (6). To test its role in cardiomyocyte cell cycle directly, we used a cardiomyocyte-specific conditional knockout (cKO) mouse and assessed DNA synthesis by thymidine analog injection at multiple time points (Fig. 1*C*). *Runx1* knockout was confirmed by excision of exon 4 from genomic DNA isolated from heart tissue compared with the ear tissue (Supplemental Fig. S1*A*). In *Runx1* cKO hearts, we observed a significant decrease in cardiomyocyte DNA synthesis at P7 compared with Myh6-CreER-control littermates (Fig. 1*D*), an observation had no effect on heart size at this same time (Supplemental Fig. S1*B*). Interestingly, we observed an increase in EdU incorporation into cardiomyocytes when assessed at P10 (Fig. 1*E*). Furthermore, when assessing total cardiomyocyte number and ploidy at P21, we observed no differences between *Runx1* cKO mice and Cre-control littermates (Fig. 1, *F* and *G*). Together, these data indicate that endogenous *Runx1* only modestly influences cardiomyocyte cell-cycle activity during postnatal development, an effect that can be recovered presumably by alternative compensatory mechanisms.

### Runx1 Is Not Required for Neonatal Heart Regeneration

During normal postnatal development when cardiomyocyte cell cycle is declining, we observed a modest and transient effect of *Runx1* knockout on cardiomyocyte cell-cycle activity, thus we wondered if a phenotype would become more apparent under heightened conditions. Within the first days of life, neonatal mice can regenerate their heart after insult (8). This regeneration process relies on cardiomyocyte proliferation to replace lost myocytes after injury (8, 11). RUNX1 expression in cardiomyocytes increases twofold at 7 days post-resection (DPR) compared with sham-operated hearts (Fig. 2*A*). To directly investigate the requirement of *Runx1* in neonatal heart regeneration, P1 apical resections were performed on *Runx1* cKO and control littermates. Cycling cardiomyocytes were labeled with EdU at 5 and 7 DPR, cardiac function was assessed at 21 DPR by echocardiography, and hearts were collected for histology at 22 DPR (Fig. 2*B*). We observed no difference in cardiomyocyte DNA synthesis in the border (Fig. 2*C*) and remote zones (Fig. 2*D*), nor in cardiac function (Fig. 2*E*), or fibrosis (Fig. 2, *F* and *G*) between *Runx1* cKO and littermate controls, indicating *Runx1* is not critically required for neonatal heart regeneration. This possibility is further supported by the observation that RUNX1-positive cardiomyocytes represent <2% of total cycling cardiomyocytes at P8, and even fewer in the context of regeneration (Supplemental Fig. S1*C*), suggesting additional mechanisms induce cardiomyocyte cell-cycle reentry.

### Runx1 Overexpression Influences Cardiomyocyte DNA Content

Although *Runx1* is not required for neonatal regeneration, our laboratory’s previous work showed that *Runx1* overexpression from birth resulted in more cardiomyocyte proliferation and an expansion of the mononuclear diploid fraction (6), a population believed to have retained proliferative competence. Therefore, we were interested in determining if the expanded mononuclear diploid pool could extend the neonatal regeneration window. To investigate this possibility, we used a mouse model whereby *Myh6-MerCreMer* drives *Runx1* overexpression (*Runx1* OE) (6). We injected tamoxifen at P0 and P1 to initiate *Runx1* OE and performed MI through permanent ligation of the left anterior descending artery on P6 mice. EdU was administered at 5 DPI, cardiac function was assessed at 28 DPI, and hearts were collected for histology the following day (Fig. 3*A*). With this tamoxifen injection regimen, we observed RUNX1 induction in ∼50% of cardiomyocytes on average (Supplemental Fig. S2, *A* and *B*). Following EdU injection at 5 DPI, we observed no difference in the number of cardiomyocyte nuclei undergoing DNA synthesis in the border and remote zones of *Runx1* OE neonates compared with Cre-positive control littermates (Fig. 3, *B* and *C*). However, EdU incorporation on its own does not indicate if proliferation or polyploidization has taken place, therefore a separate cohort of animals was used to evaluate ploidy following EdU administration. In this second experiment, tamoxifen was administered at birth, MI or sham operations were performed at P6, and EdU injections were administered at 5 DPI. Hearts were then digested into single-cell suspension at 7 DPI for cardiomyocyte nucleation and ploidy analysis. When considering all cardiomyocytes, uninjured *Runx1* OE hearts have more diploid (2 N) cardiomyocytes compared with Cre-positive control littermates (Fig. 3*D*, Supplemental Table S1) confirming our previous results (6). Following MI, *Runx1* OE hearts continue to display an increase in the diploid cardiomyocyte population compared with controls; however, the injury itself appears to increase polyploidization compared with sham in both wildtype and *Runx1* OE animals (Fig. 3*D*). Considering the EdU-positive cardiomyocytes specifically, *Runx1* OE, independent of injury status, expands the 4 N population while the 8 N population decreases in frequency (Fig. 3*E*, Supplemental Table S2). More specifically, the 1 × 4 N population expands while the tri- and tetranuclear 8 N populations decrease (Fig. 3*F*). This discrepancy across genotypes could stem from the altered starting state, whereby *Runx1* OE mice have more 1 × 2 N cardiomyocytes before injury that could become 1 × 4 N upon one round of DNA synthesis without division following injury. On the other hand, cardiomyocytes of control mice start predominantly as 2 × 2 Ns, which would instead progress to an 8 N population upon the same injury-induced DNA synthesis without division. Alternatively, the expanded EdU-positive 1 × 4 N population observed in *Runx1* OE mice could be the product of an 8 N population dividing to give rise to two 4 N daughter cells as has been described in the hepatocyte literature (12) and thus may represent cell division. Regardless, at this early time point after a P6 MI, we observe an expansion of lower ploidy classes when *Runx1* is overexpressed; however, we do not explicitly identify an EdU-positive mononuclear diploid population that would definitively represent cell division.

### Cardiomyocyte Cell Cycle Continues 1 Mo After Induction in Runx1 OE Hearts Resulting in Hyperpolyploidization

The EdU analysis performed on tissue sections in Fig. 3, *B* and *C*, captured the cell-cycle activity back from the time of injection (5 DPI). In addition, we assessed cardiomyocyte cell-cycle activity using immunofluorescence for several cell-cycle markers, which instead provides insights into any continued activity 1 mo after injury when the hearts were collected. We could not detect measurable levels of cardiomyocytes positive for Ki67, a marker of all cell-cycle phases (Fig. 4*A*), in control hearts 1 mo after injury, suggesting the response to injury was complete at this late time point. On the other hand, *Runx1* OE mice displayed a significant increase in Ki67-positive cardiomyocytes in both the border and remote zones, indicating continued cardiomyocyte cell-cycle activation (Fig. 4*B*). We next assessed cardiomyocyte expression of proliferating cell nuclear antigen (PCNA), a marker of S-phase, and again detected negligible levels in control animals, whereas *Runx1* OE littermates displayed significantly higher levels of PCNA-positive cardiomyocytes (Fig. 4, *C* and *D*). Interestingly, this phenomenon was not observed with a marker of M-phase, phospho-histone-H3 (Ph3, Fig. 4, *E* and *F*), perhaps indicating the continued cell-cycle activity is not advancing into mitosis. To assess if the observed cell-cycle activation resulted in cell division, we performed single-cell suspension EdU analysis on a separate cohort of animals, where MI was performed at P6, EdU was injected daily from 21–28 DPI, and hearts were collected by Langendorff digestion at 29 DPI (Fig. 4*G*). Hearts from sham-operated animals were also included in the analysis. We detected only a rare EdU-positive cardiomyocyte in Cre-positive control animals regardless of injury (Fig. 4*H*). On the other hand, as we observed by our in situ stains, *Runx1* OE hearts displayed substantial numbers of EdU-positive, cycling cardiomyocytes. Because EdU-positive cardiomyocytes were rare in control animals, we were not able to reliably assess their ploidy content. Instead, we were interested in how the continued cell-cycle activity in *Runx1* OE hearts impacted ploidy by comparing the ploidy state of EdU-positive cardiomyocytes labeled at 5 DPI (Fig. 3, *D* and *E*) to the ploidy state of EdU-positive cardiomyocytes labeled from 21–28 DPI (Fig. 4*G*). EdU-positive cardiomyocytes labeled 21–28 DPI presented with a dramatic expansion of the 8 N and 16 N populations (Fig. 4*I*, Supplemental Table S3). This phenomenon can be visualized more clearly by separating the data into their respective ploidy classes: considering mononuclear and binuclear cardiomyocytes, those labeled at 5 DPI were predominately 4 N (1 × 4 N and 2 × 2 N, respectively); however, the frequency of both these populations declined when labeling at 21–28 DPI and were replaced with 16 N cardiomyocyte populations (Fig. 4*J*). Together, these results indicate that *Runx1* OE drives cardiomyocyte cell-cycle activation long after wildtype animals have completed the process. However, the continued activation results in hyperpolyploidization and not further cell division.

### Persistent Runx1 OE Exacerbates Heart Function Independent of MI

**Figure 5.**
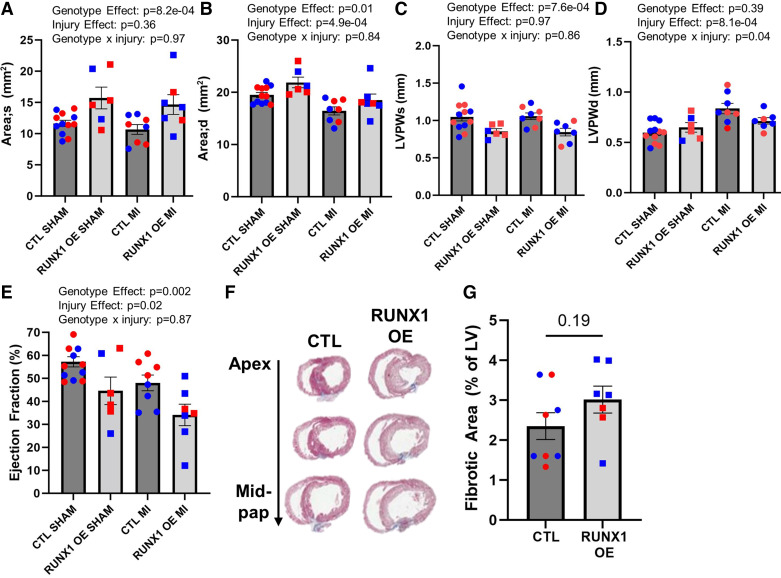
RUNX1 OE exacerbates cardiac function. *A* and *B*: quantification of ventricular luminal area in systole (*A*) and diastole (*B*) at *postnatal day 34* (P34) by B-Mode images collected with the VEVO 3100 (time line as in Fig. 3*A*). *n* = 12 Cre-positive control (Ctl) sham-operated animals, 6 RUNX1 OE sham-operated animals, 8 Cre-positive Ctl animals with myocardial infarction (MI) at P6, 7 RUNX1 OE animals with MI at P6. *P* value was assessed by two-way ANOVA, Wilks’ Lambda values for genotype vs. injury effects are reported on graph. *C* and *D*: quantification of left ventricular posterior wall (LVPW) thickness in systole (*C*) and diastole (*D*) measured from M-Mode images. *P* value is assessed by two-way ANOVA, Wilks’ Lambda values for genotype vs. injury effects are reported on graph. *E*: quantification of ejection fraction at P34. *P* value is assessed by two-way ANOVA, Wilks’ Lambda values for genotype vs. injury effects are reported on graph. *F*: representative images of hearts collected at 28 days postinjury (DPI; time line as in Fig. 3*A*), sectioned from apex to midpapillary muscle and stained for Gomori’s Trichrome Stain. *G*: quantification of fibrotic area 28 DPI, where the fibrotic area is calculated as a ratio of blue (collagen) to total area of left ventricle. *P* value is calculated by two-tailed Student’s *t* test. Data are means ± SE.

The same injury cohort used in Fig. 3 were assessed for physiological function and morphology 1 mo after MI (Fig. 3*A*). Sham-operated animals were also assessed by echocardiography. *Runx1* OE from birth results in left ventricular dilation (Fig. 5, *A* and *B*), thinning of the posterior wall (Fig. 5, *C* and *D*), and decreased ejection fraction (Fig. 5*E*), with varying influence from the injury itself. For unknown reasons, Myh6-CreER control females displayed a less severe injury compared with males as assessed by ejection fraction (Fig. 5*E*, *P* = 0.04). No other echocardiography metric displayed differences across the sexes. Considering scar, *Runx1* OE did not impact scar size 1 mo after injury (Fig. 5, *F* and *G*). These findings suggest that while *Runx1* OE does not negatively impact regeneration acutely, it does cause adverse cardiac remodeling in the long term.

### RNAseq Identifies Enhanced Hypertrophy Signaling and Suppressed p53 Signaling Following Runx1 OE

**Figure 6.**
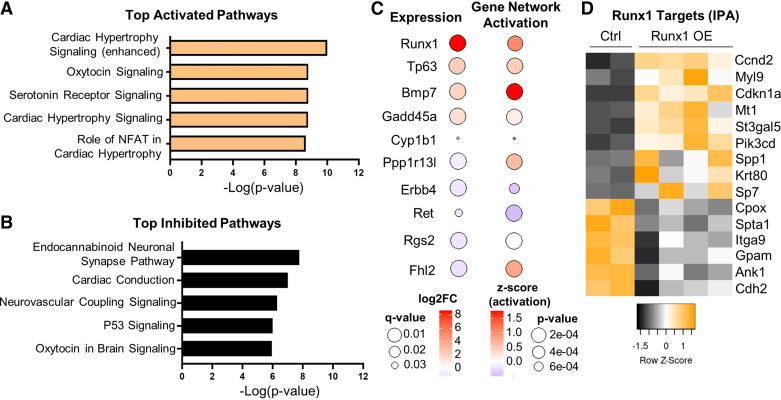
RNA sequencing identifies possible mediators of *Runx1*-induced cell-cycle activation. *A*: Ingenuity Pathway Analysis (IPA) identifying the top 5 activated pathways (based on *P* value and a positive *z*-score) from 568 differentially expressed genes (q value < 0.05) following *Runx1* OE induced at birth [*postnatal days 1* (P0) and *2* (P2) and P1 tamoxifen injections) and collection of whole ventricles at P10. *B*: IPA identifying the top 5 inhibited pathways (based on *P* value and a negative *z*-score) from 568 differentially expressed genes (q value < 0.05). *C*: bubble plot for the top 10 genes that displayed a strong gene network activation score (*P* < 10e^−4^) and were themselves also differentially expressed. Top 5 upregulated genes and top 5 downregulated genes are specifically depicted from the larger list of 15 genes (Supplemental Table S5). Bubble size indicates *q* and *P* values, whereas color indicates degree of induction. *D*: heatmap depicting the differential expression (*q* value < 0.05) of the 15 genes that contribute to the significant gene network activation score of *Runx1*. Color indicates the *z*-score between samples.

To explore the mechanisms by which *Runx1* OE influences the observed cell-cycle and functional changes, we performed transcriptomic analysis on P10 whole ventricles isolated from *Runx1 OE* (*n* = 4) and Myh6-CreER controls (*n* = 2) following tamoxifen induction at P0 and P1. Five hundred and sixty-eight genes were identified as differentially expressed (q value < 0.05) between Myh6-CreER controls and *Runx1* OE littermates (Supplemental Table S4). The differentially expressed genes were then run through Ingenuity Pathway Analysis (IPA) to identify the top activated and inhibited pathways. This analysis identified cardiac hypertrophy signaling among the top 5 activated pathways (Fig. 6*A*) and cardiac conduction and p53 signaling among the top 5 inhibited pathways (Fig. 6*B*).

To further identify potential mechanistic mediators downstream of *Runx1* that might influence cardiomyocyte cell-cycle phenotypes, we analyzed upstream regulators (genes only) of differentially expressed gene networks in IPA. We found 199 upstream regulators with a *P* value of <1 × 10^−4^ (Supplemental Table S5). Of these predicted upstream regulators, 15 were differentially expressed themselves (Fig. 6*C*), lending stronger support that these could represent direct mediators of our observed phenotypes. As expected, *Runx1* is the most strongly upregulated gene in this group (Fig. 6*C*, “Gene expression,” Expression Log Ratio 8.89, q value 2.8 × 10^−38^) and is predicted to be an upstream regulator of a strongly activated gene network (Fig. 6*C*, “Gene Network Activation Score” Activation *z*-score = 1.029, *P* value = 1.15 × 10^−4^). RUNX1 is known to serve as both a transcriptional activator and transcriptional repressor, and its direct targets are differentially expressed between Myh6-CreER control and *Runx1* OE hearts (Fig. 6*D*), contributing to its significant Gene Network Activation Score. Interestingly, of the differentially expressed direct targets of RUNX1, both CyclinD2 (*Ccnd2*), responsible for G1/S-phase cell-cycle reentry, and p21 (*Cdkn1a*), responsible for suppression of M-phase cyclins while also preventing apoptosis, were significantly upregulated in *Runx1* OE hearts (Fig. 6*D*). This seemingly contradictory combination is consistent with activation of the endomitotic cell cycle (13), an alternative cell cycle responsible for polyploidization. Finally, the upstream regulator displaying the strongest activation score, even stronger than *Runx1*, was *Bmp7* (Fig. 6*C*, activation *z*-score = 1.762, *P* value = 2.07 × 10^−5^; and expression log ratio 2.057, q value 3.07 × 10^−17^), suggesting that gene expression change in *Runx1* OE hearts could be mediated by upregulation of BMP7. BMP7 has been reported to drive cardiomyocyte cell-cycle activation in various regenerative contexts including zebrafish, neonatal cardiomyocytes, and adult mouse hearts (14).

## DISCUSSION

*Runx1* had been purported to be a marker of cardiomyocyte dedifferentiation (3, 4), a cellular process thought to coincide with cardiomyocyte proliferation. In the present study, we closely examine the effect of *Runx1* on cardiomyocyte cell cycle and proliferation using both gain- and loss-of-function mouse models. We show endogenous RUNX1 expression in cardiomyocytes only modestly influences cardiomyocyte cell-cycle activity in the context of postnatal development, a result that corrects itself later and does not extend to neonatal heart regeneration. In addition, in a wild-type C57Bl/6J heart, only a rare Ki67-positive cardiomyocyte also expresses RUNX1, suggesting other mechanisms are responsible for cardiomyocyte cell-cycle activation and weaken the argument that RUNX1 is a marker of cardiomyocyte dedifferentiation events. Together these observations suggest *Runx1* is not required for cardiomyocyte proliferation.

Regarding our *Runx1* cKO studies, we acknowledge some weaknesses in our experimental design. RUNX1 expression in cardiomyocytes is rare and only a brief snapshot of activity is recorded by EdU injection, which labels just ∼1–2 h (15). Thus, it remains possible our narrow analysis window missed a more relevant time point. Furthermore, while our developmental studies in Fig. 1 controlled for Cre recombinase, our cKO P1 ventricular resection experiments did not. Cre alone is able to drive cardiomyocyte cell-cycle activation (16), therefore cKO animals may display heightened cell-cycle activity stimulated by Cre, which is then reduced back to control levels by loss of *Runx1*. In other words, it remains possible that the two alleles cancel each other out. Regardless, the number of RUNX1-positive CMs postinjury is modest. Thus, should we have missed a cell-cycle effect it was unlikely to influence the physiological outcomes as we report here, and this is in line with previous reports (9).

On the other hand, cardiomyocyte-specific overexpression of RUNX1 in the neonatal context can significantly drive cell-cycle activity; however, the effect of this cell-cycle induction results in dramatically different outcomes depending on when cycling cells are labeled. For example, Swift et al. (6) labeled cycling cardiomyocytes during the first postnatal week and observed clear evidence of cardiomyocyte proliferation (EdU-positive, mononuclear, and diploid cardiomyocytes). In our current study, we were able to recapitulate the expansion of the diploid cardiomyocyte population with *Runx1* induction at birth; however, our delayed EdU regimen to 5 DPI hearts (equivalent to P11) failed to identify definitive proliferative events as Swift et al. (6) had seen. Nevertheless, the EdU-positive cardiomyocyte population in *Runx1* OE hearts labeled at 5 DPI had significantly lower total DNA content compared with controls. This phenomenon could be explained by altered starting states or unconventional examples of cell division as has been seen in hepatocytes (12). We observe the most striking differences when labeling cycling cardiomyocytes at 21–28 DPI. In this final assessment, we note that the cardiomyocytes in control injured animals have overwhelmingly ceased cycling; however, *Runx1* OE cardiomyocytes continue to cycle. However, *Runx1* OE cycling cardiomyocytes appear no longer competent to divide, resulting in severe hyperpolyploidization. Together, these results suggest *Runx1* is sufficient to drive cardiomyocyte cell-cycle activation, which may for a time result in proliferation, but when expression is protracted long term results in endomitosis.

To our knowledge, there are two previous studies that have directly examined the effect of *Runx1* in cardiomyocytes in a postinjury context. McCarroll et al. (17) concluded that RUNX1 expression in cardiomyocytes resulted in eccentric hypertrophy and adverse cardiac remodeling following adult MI. Furthermore, cardiomyocyte-specific knockout protected from these adverse effects (17). Our experiments of conditional knockout animals did not demonstrate the same beneficial effects; however, our overexpression studies may agree with their conclusions. Notably, by immunofluorescence, we only observe a rare cardiomyocyte that expresses RUNX1, which might explain why the loss of *Runx1* has little physiological effect in our experiments. Of course, the injury context, adult MI in McCarroll et al. (17) compared with neonatal ventricular resection in our present study, are completely different, thus perhaps not comparable. Swift et al. (6) examined the effect of *Runx1* overexpression on cardiomyocyte ploidy and proliferation in the context of postnatal development and in the context of adult MI. Swift et al. observed increased proliferation with analysis 1 wk after injury, which coincided with a transient reduction in scar size and transient functional benefit. Notably, these benefits were lost by 1-mo post-MI. Our present study agrees with these findings; however, our ploidy analysis at a later postinjury time point unveils that the continued expression of RUNX1 eventually contributes to severe hyperpolyploidy, ventricular dilation, and impaired contractility.

The RNAseq analysis provides insights into the transcriptional changes associated with the cell-cycle and physiological phenotypes we observe. With induction of RUNX1 expression from birth, P10 *Runx1* OE hearts display enhanced hypertrophic signaling, suppressed P53 signaling, and suppressed cardiac conduction signaling compared with Cre controls. Repressed cardiac conduction signaling may be in alignment with McCarroll et al. (17) who reported improved calcium handling in *Runx1* cKO hearts. As anticipated, *Runx1* was the most strongly differentially expressed gene and was a predicted upstream regulator of one of the most strongly activated gene networks. Among its widely reported direct targets are *Ccnd2* and *Cdkn1a* (p21) (18), both of which are specifically upregulated in *Runx1* OE hearts. Considering the canonical mitotic cell cycle, these two genes can have contrasting roles; however, in the case of further polyploidization, as we see here, upregulation of both targets is consistent with endomitosis, where *Ccnd2* can drive G1/S activation while upregulation of *Cdkn1a* suppresses M-phase thereby blocking full cell division. Upstream regulator analysis also identified *Bmp7* as a candidate regulator of a strongly activated gene network. Notably, *Bmp7* is a direct target of *Runx1* in other developmental contexts (19), though it is not among the genes identified by IPA *Runx1* Gene Network Activation. *Bmp7* has been identified as a growth factor that is both necessary and sufficient to drive cell-cycle activation in cardiomyocytes across a variety of species (14). Collectively, these data suggest that *Runx1* OE is likely driving cell-cycle activation through both direct and indirect means, including possibly via BMP7 upregulation.

The field of heart regeneration has been hyperfocused on driving cardiomyocyte proliferation. Many studies conflate any cardiomyocyte cell-cycle activation with proliferation; however, it is becoming more and more evident that much of the cell-cycle activation is in fact contributing to further polyploidization rather than proliferation (20, 21). Here, we observed that continued RUNX1 expression resulted in extended cell-cycle activation and DNA synthesis but not increased M-phase activity. Furthermore, our single-cell suspension analysis revealed that cardiomyocytes still cycling 1 mo after injury are specifically becoming hyperpolyploid. Physiologically, these hearts display attributes of dilated cardiomyopathy or adverse remodeling. Other studies targeting independent gene networks that influence cardiomyocyte polyploidy have observed similar effects on heart function when cardiomyocyte ploidy is increased (9, 22–26). Although each finding is correlative on its own, taken together these observations raise the concern that prolonged stimulation of cell cycle in cardiomyocytes may have adverse effects. Furthermore, if initial cardiomyocyte proliferation is required to stimulate a regenerative response, strategies to induce cell-cycle activation transiently are likely warranted.

## DATA AVAILABILITY

Source RNAseq data for this study are available at Gene Expression Omnibus Accession No. GSE266578.

## SUPPLEMENTAL DATA

10.6084/m9.figshare.25769037.v1 Supplemental Figs. S1 and S2 and Supplemental Tables S1–S5: https://doi.org/10.6084/m9.figshare.25769037.v1.

## GRANTS

This work was funded by National Institutes of Health (NIH) Grants R01HL155085 (to M.P.), 3R01HL155085-S1 (to M.P. and K.A.A.), F31HL162468 (to S.K.S), F32HL150958 (to M.A.F), and R01HL141159 and R01HL170547 (to C.C.O). T. Buddell was supported by NIH Grant T32HL134643, and A. L. Purdy was supported by NIH Grant T32HL007852. Vevo 3100 echocardiography machine was purchased and maintained by NIH Grant S10OD025038.

## DISCLOSURES

No conflicts of interest, financial or otherwise, are declared by the authors.

## AUTHOR CONTRIBUTIONS

K.A.A., S.K.S., C.C.O., and M.P. conceived and designed research; K.A.A., M.A.F., S.K.S., S.V.C., A.L.P., T.B., M.E.K., K.G.A., S.P., and S.L.B. performed experiments; K.A.A., M.A.F., S.K.S., S.V.C., A.L.P., T.B., M.B.V., and M.P. analyzed data; K.A.A., M.A.F., S.K.S., M.B.V., C.C.O., and M.P. interpreted results of experiments; K.A.A., M.B.V., and M.P. prepared figures; K.A.A. and M.P. drafted manuscript; K.A.A., M.A.F., S.K.S., C.C.O., and M.P. edited and revised manuscript; K.A.A., S.L.B., M.A.F., S.K.S., S.V.C., A.L.P., T.B., M.E.K., K.G.A., S.P., M.B.V., C.C.O., and M.P. approved final version of manuscript.
